# BioRT‐HBV 1.0: A Biogeochemical Reactive Transport Model at the Watershed Scale

**DOI:** 10.1029/2024MS004217

**Published:** 2024-11-30

**Authors:** Kayalvizhi Sadayappan, Bryn Stewart, Devon Kerins, Andrew Vierbicher, Wei Zhi, Valerie Diana Smykalov, Yuning Shi, Marc Vis, Jan Seibert, Li Li

**Affiliations:** ^1^ Department of Civil and Environmental Engineering The Pennsylvania State University University Park PA USA; ^2^ Department of Plant Science The Pennsylvania State University University Park PA USA; ^3^ Department of Geography University of Zurich Zurich Switzerland

**Keywords:** water quality, biogeochemistry, hydrology, reactive transport modeling, carbon, nutrient processes

## Abstract

Reactive Transport Models (RTMs) are essential tools for understanding and predicting intertwined ecohydrological and biogeochemical processes on land and in rivers. While traditional RTMs have focused primarily on subsurface processes, recent watershed‐scale RTMs have integrated ecohydrological and biogeochemical interactions between surface and subsurface. These emergent, watershed‐scale RTMs are often spatially explicit and require extensive data, computational power, and computational expertise. There is however a pressing need to create parsimonious models that require minimal data and are accessible to scientists with limited computational background. To that end, we have developed BioRT‐HBV 1.0, a watershed‐scale, hydro‐biogeochemical RTM that builds upon the widely used, bucket‐type HBV model known for its simplicity and minimal data requirements. BioRT‐HBV uses the conceptual structure and hydrology output of HBV to simulate processes including advective solute transport and biogeochemical reactions that depend on reaction thermodynamics and kinetics. These reactions include, for example, chemical weathering, soil respiration, and nutrient transformation. The model uses time series of weather (air temperature, precipitation, and potential evapotranspiration) and initial biogeochemical conditions of subsurface water, soils, and rocks as input, and output times series of reaction rates and solute concentrations in subsurface waters and rivers. This paper presents the model structure and governing equations and demonstrates its utility with examples simulating carbon and nitrogen processes in a headwater catchment. As shown in the examples, BioRT‐HBV can be used to illuminate the dynamics of biogeochemical reactions in the invisible, arduous‐to‐measure subsurface, and their influence on the observed stream or river chemistry and solute export. With its parsimonious structure and easy‐to‐use graphical user interface, BioRT‐HBV can be a useful research tool for users without in‐depth computational training. It can additionally serve as an educational tool that promotes pollination of ideas across disciplines and foster a diverse, equal, and inclusive user community.

## Key Points

- We introduce BioRT‐HBV, a watershed scale reactive transport model that is parsimonious, flexible with reaction network, easy to use and requires minimal data
- BioRT‐HBV can simulate a variety of user‐defined biogeochemical processes, including carbon and nitrogen processes
- BioRT‐HBV is open source for any researchers interested in ecohydrological and biogeochemical reactive transport processes

## 1 Introduction

Hydrological and biogeochemical processes occurring in a watershed collectively shape the timing, magnitude, and quality of water in streams and rivers. These processes are driven by external hydroclimatic forcings and human perturbations, and modulated by watershed characteristics including soil structure, lithology, vegetation cover, land use, and topography (Li et al., 2021, 2022; McDowell et al., 2023; Sullivan et al., 2022). It is essential to understand interacting hydrological and biogeochemical processes and to forecast river water response to future climate and human perturbations (Duffy et al., 2014; Li et al., 2024).

A variety of models have been developed to understand water quality and eco‐hydrological interactions. Examples include SWAT (Soil Water Assessment Tool) (Hu et al., 2007; Luo & Zhang, 2009; Rajib et al., 2020), HSPF (Hydrological Simulation Program – FORTRAN) (Filoso et al., 2004; Laroche et al., 1996), HYPE (Hydrological Predictions for the Environment) (Arheimer et al., 2012; Veinbergs et al., 2017), MIKE SHE (Système Hydrologique Européen) (Hou et al., 2021; Jaber & Shukla, 2012), INCA (INtegrated CAtchment model) (Bastrup‐Birk & Gundersen, 2004; Wade et al., 2002), and VELMA (Visualizing Ecosystem Land Management Assessments) (Abdelnour et al., 2011, 2013; Halama et al., 2024; Johnston et al., 2024). For example, the model VELMA, a spatially explicit eco‐hydrological model with three components (hydrology, soil temperature, and biogeochemistry), simulates the fate and transport of nitrogen species, organic carbon and contaminants in a catchment. The model simulates carbon and nitrogen storage and their cycling including plant‐soil interactions such as plant mortality and plant uptake of nutrients, organic material decomposition, atmospheric deposition, nitrification and denitrification.

Most of these models focus on a limited number of reactions and solutes defined a priori and offer limited flexibility for modifying included reactions and introducing new reactions or solutes. As an example, the PULSE model – the first water quality model based on HBV (Hydrologiska Bryåns Vattenavdelning) hydrological model – can simulate variations in stream pH and alkalinity alone (Bergström et al., 1985). The PULSE model was later augmented to simulate the transport of the conservative tracer ^18^O (Lindström & Rodhe, 1986) and inorganic nitrogen (Bergström et al., 1987; Brandt, 1990). Nitrogen (N) processes related to nitrogen transformation in streams, lakes and wetlands were further added, ultimately leading to the development of the HBV‐N model (Arheimer & Brandt, 1998; Arheimer & Wittgren, 1994; Pettersson et al., 2001). This model evolved into HBV‐NP with the inclusion of transport and transformation processes of soluble and particulate phosphorus (P) (Andersson et al., 2005; Arheimer et al., 2005). Based on HBV‐NP, the Hydrological Predictions for the Environment (HYPE) model was developed for simulations at high spatial resolution (Lindström et al., 2010). The HYPE model has been further modified to simulate organic carbon processes (Pers et al., 2016). The model can now simulate carbon and nutrient processes but still lacks the capability to model other solutes such as cations and anions mobilized by chemical weathering.

Biogeochemical reactions are complex and often cover a wide range of solutes and processes, varying from abiotic reactions such as chemical weathering to biotic reactions such as soil respiration, nutrient transformation, and plant uptake of nutrients. Users often do not know which reactions play a predominant role in determining the dynamics of a particular nutrient or solute a priori. Thus, there is a need for generic, flexible models where users themselves can define the types of reactions and solutes and test different combinations of reactions as hypotheses to determine which reactions most influence solute dynamics. Multi‐component Reactive Transport Models (RTMs) have formulated an approach to do this since 1980s and can serve this purpose (Lichtner, 1985, 1988). These models solve reactive transport equations for a variety of user‐defined solutes based on reaction stoichiometry and thermodynamics and kinetics defined in a generic database and input files (Steefel et al., 2015; Steefel & MacQuarrie, 1996). Traditional RTMs have primarily focused on subsurface processes (Li, Maher, et al., 2017; Lichtner, 1988). Recent watershed‐scale RTMs such as BioRT‐Flux‐PIHM and Amanzi‐ATS have evolved to include hydrometeorological conditions, land surface processes and their interactions with subsurface processes at the watershed scale (Bao et al., 2017; Coon et al., 2019; Jan et al., 2021; Molins et al., 2022; Stolze et al., 2024; Wen et al., 2024; Zhi et al., 2022). The emergent watershed‐scale RTMs have facilitated our understanding of complex hydrological and biogeochemical coupling. However, characterized by spatial and computational complexity, these models require extensive field measurements that are often only available in intensively monitored catchments. Their computational complexity also acts as a barrier to users without computational expertise. There is a pressing need for flexible and parsimonious modeling tools that are accessible to users from diverse backgrounds without extensive computational training (Perdrial et al., 2023; Singha et al., 2024).

In this context, we developed BioRT‐HBV model, which integrates the widely‐used hydrologic model HBV with BioRT, the biogeochemical module of BioRT‐Flux‐PIHM (Zhi et al., 2022). HBV is a semi‐distributed, bucket‐type hydrological model that has been used to simulate watershed‐scale hydrological processes in about 100 countries (Bergström, 1992; Seibert & Bergström, 2022). It originated in the 1970s (Bergström, 1992; Bergström & Forsman, 1973) and now has several different versions for different purposes (Bergström & Lindström, 2015). The driving philosophy of HBV has been a simple but robust model with minimal data requirements (Lindström et al., 1997). Several software implementations of HBV model exist, of which HBV‐light was chosen as it offers a user‐friendly graphical user interface (GUI) and has been widely used for both educational and research purposes (Seibert & Vis, 2012). The simulated results from HBV‐light would be rather similar to results from other implementations of the HBV model. We use the term “HBV” model hereafter to mean the “HBV‐light” implementation of the HBV model.

**Figure 1:**
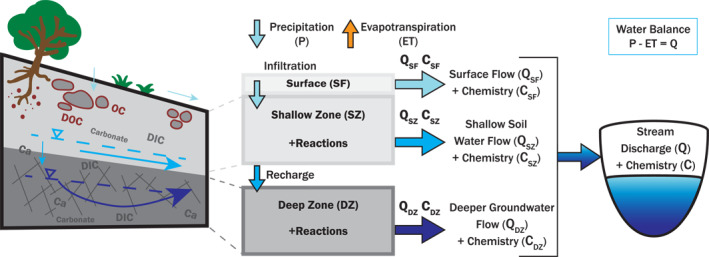
A conceptual diagram of BioRT‐HBV model structure. Note that the shallow zone (SZ) and the deep zone (DZ) correspond to the upper and lower zones in HBV respectively. BioRT‐HBV has an additional surface zone (SF) for simulating potential reactions on or near ground surface as surface flow occurs, mobilizing solutes and solids from the ground surface to streams. The *Q*_SF_, *Q*_SZ_, and *Q*_DZ_ correspond to the “effective” flows defined in HBV as *Q*_0_, *Q*_1_, and *Q*_2_, respectively. The BioRT‐HBV model can incorporate input precipitation chemistry and simulate distinct biogeochemical processes in the three “effective” zones based on user‐defined reaction network. Note: We use HBV‐light version of HBV model with “standard” structure and three lateral flows.

BioRT‐HBV model reproduces catchment‐scale solute dynamics by simulating biogeochemical reactions in three zones which contains the three major water flow pathways (Figure 1). BioRT‐HBV uses the hydrological output from HBV, including soil moisture, water storage, and flow as drivers. It uses reaction thermodynamics and kinetics in simulating reaction rates similar to traditional RTMs widely used in the geochemistry community; it solves mass conservation equations for solute concentrations in water by integrating advective transport and reaction processes (Lichtner, 1988; Steefel et al., 2015; Steefel & MacQuarrie, 1996). BioRT‐HBV can model a variety of biogeochemical processes including chemical weathering and soil respiration, nutrient transformation, and sediment mobilization. The code is written generically to allow users to define the system of reactions and solutes and solids they intend to simulate in input files. Its extensive database defines reaction stoichiometry, thermodynamics, and kinetics, and can be extended by users. Here we present the model description, governing equations, and numerical scheme. We additionally show example applications of BioRT‐HBV in simulating watershed‐scale reactive transport processes in a headwater catchment in the Sleepers River Research Watershed in Vermont, USA.

## 2 Model Description

### 2.1 Model Structure

The HBV model has been well documented in literature (Bergström, 1992; Seibert & Bergström, 2022; Seibert & Vis, 2012). Here we briefly describe its “standard” model structure that is necessary to understand the structure of BioRT‐HBV. HBV simulates hydrological processes, including evapotranspiration and streamflow generation. The model includes two subsurface zones, the upper and lower zones (UZ and LZ), which we conceptually consider as corresponding to the shallow soil zone (SZ) and deeper groundwater zone (DZ) in BioRT, respectively (Figure 1). The shallow zone represents the shallow, often unsaturated subsurface where water table and lateral water flow are transient and where the water table can rise to the ground surface under very wet conditions (Sullivan et al., 2016; Torres et al., 2015). This is the zone where water comes and goes quickly and interacts with weathered minerals and organic matter in soil. We consider the soil moisture routine of HBV as part of the shallow zone. The deep zone represents the deeper subsurface that is generally saturated and provides baseflow. Conceptually this can represent shallow aquifers below the soil zone where older water is in contact with partially weathered or parent bedrock (Anderson et al., 1997; Frisbee et al., 2013). Note that while the real‐world system consists of innumerable number of zones and flow paths, the model uses “effective” representations to distill the complexity into a form that captures the essential hydro‐biogeochemical functioning of a watershed.

In the HBV model, streamflow *Q* is generated as the sum of three flow components, *Q*_0_, *Q*_1_, and *Q*_2_, representing different flow pathways through which water drains into rivers and streams. The upper zone storage generates *Q*_0_ and *Q*_1_, and the lower zone generates *Q*_2_. There is no explicit surface runoff or flow in the HBV model. *Q*_0_ is considered as “quick” flow that occurs from the upper zone when water storage exceeds a threshold. In BioRT‐HBV, we consider *Q*_0_ as rapid surface or overland flow that occurs under very wet conditions. To assign physical interpretations to the three flow components, we changed the HBV terminology in BioRT‐HBV (Figure 1). We use *Q*_SF_ for *Q*_0_, conceptually representing rapid surface flow or quick flow; *Q*_SZ_ for *Q*_1_, representing lateral flow from the shallow soil zone; and *Q*_DZ_ for *Q*_2_, representing flow from the deep groundwater zone.

An additional surface zone (SF) was added in BioRT‐HBV to represent the transient above‐ground water storage that flows as rapid surface flow *Q*_SF_ (or *Q*_0_ in HBV terminology), mobilizing solute fluxes at or near ground surface to streams under very wet conditions. Note that while *Q*_SF_ (or *Q*_0_) occurs from the upper zone in HBV, we consider it to occur from SF in BioRT‐HBV. We assume that the water entering the surface zone as *Q*_SF_ has the same chemistry as the water infiltrating into the shallow zone, determined by the mixing of rainfall and snowmelt. In other words, reactions occurring in the surface zone do not influence the chemistry of the infiltrating water because this quick infiltrating water has little time to react with surface materials. On the other hand, surface runoff can interact with the ground surface and top subsurface layers such as surface organic matter and litter when it flows laterally, and mobilize fine sediments (soil erosion), ashes, road salts and nutrients on its way toward streams. These reactions influence the chemistry of the surface runoff or *Q*_SF_ reaching the stream. Users can decide whether or not surface reactions are simulated in the model. Each zone is considered as a well‐mixed reactor in the model.

Reactions are not simulated in snowpack. The chemistry of incoming snow is defined by precipitation chemistry in the user input files. The model keeps track of snow chemistry based on mass balance of solutes in snow water storage. Snowmelt has the same chemistry as snowpack at every time step. HBV calculates soil moisture and “dynamic” water storage that generates streamflow in the upper and lower zones. The shallow zone water storage in BioRT‐HBV includes dynamic water storage in the upper zone and the soil moisture storage, while the deep zone storage includes the dynamic water storage in the lower zone. An additional water storage termed “passive water storage” is included in each subsurface zone – SZ and DZ. The passive water storage represents the water that does not contribute to streamflow generation but participates in evapotranspiration, solute transport, and reactions. These water storages are not explicitly modeled in the HBV model, and we assign these values for the shallow and deep zones in BioRT input files. The signature of this storage is often reflected in tracer transport and stream chemistry and therefore can be calibrated using tracer and stream chemistry data. All water fluxes and storages except for passive water storages are from the HBV model, either directly from its output file or back calculated using HBV parameters in its parameter file – both of which serve as inputs to BioRT‐HBV model.

The relative contributions of *Q*_SF,_ *Q*_SZ_ and *Q*_DZ_, to total stream discharge depend on hydroclimate forcings and the land and subsurface characteristics. They are quantified by calibrating the model parameters in HBV that characterize how water moves through a watershed, including, for example, how much water infiltrates to the shallow zone and recharges (or percolates as it often is called in the HBV model descriptions) to the deep zone to become deeper groundwater. Note that these three flows are simplified representations of the innumerable flow paths in natural systems. Such simplification is necessary as we do not have the data and computational luxury to fully represent these details.

### 2.2 Governing Equations

Multi‐component reactive transport systems usually have multiple solute species participating in various reactions. Following RTM tradition (Lichtner, 1988; Steefel & Lasaga, 1994; Steefel & MacQuarrie, 1996), BioRT‐HBV solves the governing equations for each zone for the primary species. The primary species are the building blocks of the chemical system; the secondary species can be expressed by the concentrations of primary species via equilibrium reactions and laws of mass action. The model solves differential equations for the concentrations of primary species, based on which the concentrations of secondary species can be calculated. This approach eliminates the need to solve for all species via time stepping. Here we write the representative equations for an arbitrary primary species *i* within a total number of *n* primary species in each zone.

In the surface zone (SF):

(1) $$\frac{d(C_{\text{SF},i}V_{w,\text{SF}})}{dt}=P_{\text{rain}}C_{\text{rain},i}+Q_{\text{snowmelt}}C_{\text{snowmelt},i}-Q_{\text{SF}}C_{\text{SF},i}-Q_{\text{infil}}C_{\text{infil},i}+R_{\text{SF}},i=1,\ldots ,n$$

where *C*_infil,*i*_ (mol/L) is the concentration of solute *i* in the infiltrating water, determined by the mixing of rainwater and snowmelt water. Concentrations in infiltrating water are calculated as follows: ${\;C}_{\text{infil},i}=\frac{P_{\text{rain}}C_{\text{rain},i}+Q_{\text{snowmelt}}C_{\text{snowmelt},i}}{P_{\text{rain}}+Q_{\text{snowmelt},i}}$. Snow can accumulate at the ground surface, and its chemistry is kept track of using the equation: $\frac{d(C_{\text{snow},i}V_{w,\text{snow}})}{dt}=P_{\text{snow}}C_{\text{snow},i}-Q_{\text{snowmelt}}C_{\text{snowmelt},i}$.

In the Shallow Zone (SZ):

(2) $$\frac{d\left(C_{\text{SZ},i}V_{w,\text{SZ}}\right)}{dt}=Q_{\text{infil}}C_{\text{infil},i}-\left(Q_{\text{SZ}}+Q_{\text{perc}}\right)C_{\text{SZ},i}+R_{\text{SZ}},i=1,\ldots ,n$$

In the Deep Zone (DZ):

(3) $$\frac{d\left(C_{\text{DZ},i}V_{w,\text{DZ}}\right)}{dt}=Q_{\text{perc}}C_{\text{SZ},i}-Q_{\text{DZ}}C_{\text{DZ},i}+R_{\text{DZ},i\;},i=1,\ldots ,n$$

where *P*_snow_ (mm/day) is the precipitation falling as snow, *P*_rain_ (mm/day) is the precipitation falling as rainfall, *Q*_snowmelt_ (mm/day) is the flow from snowmelt, *Q*_infil_ (mm/day) is the water infiltrating into the shallow zone, *Q*_perc_ (mm/day) is the water percolating (or recharge) from the shallow zone to the deep zone. Here *V*_*w,*SF_, *V*_*w,*SZ_, and *V*_*w,*DZ_ (mm) are the water storages in surface zone, shallow zone, and deep zone, respectively, and *V*_*w,*snow_ (mm) is the water storage in snowpack. Water volumes (*V*_*w,*snow_, *V*_*w,*SF_, *V*_*w,*SZ_ and *V*_*w,*DZ_) and water fluxes (*Q*_snowmelt_, *Q*_infil_, *Q*_SF_, *Q*_SZ_, *Q*_DZ_ and *Q*_perc_) are drainage‐area‐normalized values (outputs from the HBV model) and therefore have units of mm (volume per unit drainage area) and mm/day of water respectively. *C*_ppt*,i*_, *C*_snow*,i*_, *C*_infil,*i*_, *C*_SF*,i*_, *C*_SZ*,i*,_ and *C*_DZ*,i*_ (mol/L) are the concentrations of solute *i* in precipitation, snowpack, infiltrating water entering the shallow zone, surface zone, shallow zone, and deep zone, respectively. The reaction rates *R*_SF*,i*_, *R*_SZ*,i*_, and *R*_DZ*,i*_ (mol/m^2^/day) are those of solute *i* in surface zone, shallow zone, and deep zone, respectively. Since water fluxes and storage are area‐normalized, the reaction rates are also area normalized. If a solute participates in more than one reaction, the rate of each reaction can be spelled out separately. The *R* terms in Equations (1), (2), (3) are the summation of multiple reaction rates in each zone, as exemplified later in the applications.

BioRT‐HBV solves for solute concentrations at regularly spaced time steps (seconds, hours, or days). Time step is user‐defined in the input files (details in “2.5 Model setup and input/output”). Note that for simplicity we used the time unit of “day” in the above equations. Unit conversation is done in the code when other time steps are used to maintain consistent mass conservation. A Graphical User Interface (GUI) is available for the model in addition to the command line interface.

### 2.3 Reactions

BioRT‐HBV can simulate a variety of reactions including chemical weathering (e.g., mineral dissolution and precipitation), microbial and root respiration reactions, nutrient transformation, ion exchange, surface complexation, among others along with the influence of environmental conditions on these reactions. Users can define the types of reactions and solutes to be included in the reaction network, and the form of reaction rate laws.

#### 2.3.1 Reaction Rate Dependence on Environmental Conditions

The rates of reactions in subsurface generally depend on temperature, soil moisture, and water table level (Davidson et al., 2004; Davidson & Janssens, 2006). In other words, reaction rates depend not only on the properties of reacting materials or substrate, but also on environmental conditions. We therefore use rate laws that reflect rate dependence on these conditions:

(4) $$r=kA\;f\left(T\right)f\left(S_{w}\right)f\left(Z_{w}\right)$$

where *r* is the reaction rate (mol/m^3^/sec), *k* is the reaction rate constant (mol/m^2^/sec), *A* is the material surface area abundance (m^2^/m^3^) and *f*(*T*), *f*(*S*_*w*_), and *f*(*Z*_*w*_) describe the rate dependence on temperature (*T*), soil moisture (*S*_*w*_), and water table levels (*Z*_*w*_), respectively, as detailed below.

***Temperature dependence function f(T)*:** Biotic reaction rates typically increase with temperature. Here we use the *Q*_10_‐based approach that has been widely adopted (Davidson & Janssens, 2006; Elberling, 2005):

(5) $$f\left(T\right)=Q_{10}^{\left|T-20\right|/10}$$

where *Q*_10_ is the temperature coefficient representing the relative increase in rates when temperature increases by 10°C, and *T* is temperature (°C). If *Q*_10_ is 1, *f*(*T*) becomes 1 such that the rate has no dependence on temperature.

***Soil moisture dependence function f(S***_***w***_ ***)*:** Microbial respiration rates typically increase with soil moisture until some intermediate value, beyond which the rates decrease as they change from substrate limited to oxygen limited (Or et al., 2007). Similarly, root respiration rates also peak at some intermediate soil moisture conditions. To simulate this dependence on soil moisture, BioRT‐HBV uses the following generic dependence function (Yan et al., 2018):

(6) $$f\left(S_{w}\right)=\left\{\begin{array}{c} {\left(\frac{S_{w}}{S_{w,c}}\right)}^{n},\text{when}\;S_{w}\leq S_{w,c}\\ {\left(\frac{1-S_{w}}{1-S_{w,c}}\right)}^{n},\text{when}\;S_{w}>S_{w,c} \end{array}\right.$$

where *S*_*w*_ is the soil moisture, *S*_*w,c*_ is the critical soil moisture at which reaction rates peak, and *n* is the soil moisture dependence exponent. The term *S*_*w,c*_ indicates that increasing moisture content does not always translate to higher rates. For example, O_2_ levels become low under water‐saturated conditions, limiting the relatively fast aerobic reactions. The slower anaerobic reactions become dominant then, reducing the overall rates of biogeochemical reactions (Schlesinger & Bernhardt, 2020). Under rare conditions where rates do not depend on soil moisture, *n* can be set as zero to disable the soil moisture dependence. This function can also be used for abiotic weathering reactions. Weathering rates typically increase with water content as the wetted mineral surface area increases with increasing soil moisture (Li, Bao, et al., 2017). For weathering reactions, the *S*_*w,c*_ value can be set as one or close to one so that the weathering rates almost always increase with water content.

***Water table depth dependence function f(Z***_***w***_ ***)*:** In addition to soil moisture, water table depth and the depth distribution of solute sources can further influence reaction rates and solute mobilization (Seibert et al., 2009; Zhi & Li, 2020). For example, organic matter is typically more abundant at shallow depths (Souza et al., 2023) such that shallow water tables can often access more organic matter and mobilize dissolved organic carbon (DOC) that is sorbed on soil surface to stream. A rising water table can also promote lateral and vertical hydrological connectivity and enhance reaction rates (Clow & Mast, 2010; Xiao et al., 2021). On the other hand, falling water table in peat lands have been associated with elevated soil respiration and carbon losses (L. Ma et al., 2022). BioRT‐HBV uses an exponential function to account for the water table depth dependence following practices in literature (Bai et al., 2016; Ottoy et al., 2016; Seibert et al., 2009; Zhi et al., 2022).

(7) $$f\left(Z_{w}\right)=\exp\left(-\alpha {Z_{w}}^{\beta }\right)$$

where *Z*_*w*_ is the water table depth, *α* and *β* are the parameters that determine the magnitude and direction of the dependence on water table depth respectively. When *α* = 0, the rates have no dependence on water table depth. When *β* = 1, the rate increases as water table depth decreases (water table rises); when *β* = −1, the rate decreases as water table depth decreases. In input files, the product of these parameters (α*β), rather than their absolute values, are prescribed.

#### 2.3.2 Rates of Weathering Reactions

For abiotic reactions like weathering, the rates generally follow the classic Transition State Theory (TST) (Aagaard & Helgeson, 1982; Helgeson et al., 1984; Lasaga, 1984; Steefel et al., 2015), which prescribes reaction rates as dependent on mineral properties, concentrations of catalyzing solutes, and how far away the reaction is from equilibrium:

(8) $$r=kA_{\text{mineral}}a^{m}\left(1-\frac{\text{IAP}}{K_{\text{eq}}}\right)$$

Here *r* is the reaction rate (mol/m^3^/s), *A*_mineral_ is the wetted mineral surface area per unit volume (m^2^/m^3^), *k* is the kinetic reaction rate constant of the reaction (mol/m^2^/s), *K*_eq_ is the equilibrium constant of the reaction, “a” represents the activity (equals to concentration in most natural inland waters) of a solute that can catalyze or inhibit weathering, and the exponent “m” represents the extent of dependence on the solute concentration. For example, hydrogen ion (H^+^) often accelerates weathering and the reaction rates depend on pH. The ratio IAP (Ion Activity Product)/*K*_eq_ describes how far away the reaction is from equilibrium. Note that we use the traditional units and way of writing equations for reaction rates here. Unit conversation is done in the code while solving for advective transport and reaction equations to maintain mass conservation and ensure unit consistency.

#### 2.3.3 Rates of Biology‐Mediated Reactions

Microbe‐mediated redox reactions can be limited by the concentrations of electron donors (e.g., dissolved organic carbon) and/or electron acceptors. This is often the case in the deeper subsurface where organic materials are less reactive and electron acceptors such as O_2_ are limited. In that case, the reaction rates follow the Monod form (Monod, 1949).

(9) $$r=kA\prod_{ii=1}^{mm}\left(\frac{C_{ii}}{C_{ii}+K_{M,ii}\;}\right)$$

where *r* is the reaction rate (mol/m^3^/s), *k* is the kinetic reaction rate constant of the reaction (mol/m^2^/s), *A* is the material surface area abundance (m^2^/m^3^), *K*_*M,ii*_ is the half saturation coefficient (mol/L) of electron donor or acceptor ii and mm is the total number of electron donors and acceptors that are limiting.

In addition, when multiple electron acceptors coexist, the redox reactions occur in a sequence following the biogeochemical redox ladder (Schlesinger & Bernhardt, 2020). This can be accomplished by including inhibition terms in the following form (Li, 2019; Van Cappellen & Gaillard, 1996).

(10) $$r=kA\prod_{ii=1}^{mm}\left(\frac{C_{ii}}{C_{ii}+K_{M,ii}\;}\right)\prod_{j=1}^{nn}\left(\frac{K_{I,j}}{C_{j}+K_{I,j}\;}\right)$$

Here *K*_*I,j*_ is the inhibition coefficient (mol/L) of inhibitor *j* and *nn* is the total number of inhibitors. As an example, for reactions that use nitrate as the electron acceptor, the inhibition term can include the concentration of O_2_ as an inhibitor, as O_2_ has to be almost depleted for denitrification to become dominant. Similarly, for reactions where iron oxide is the electron donor, both O_2_ and NO_3_ can be inhibitors with their own respective inhibition coefficient.

These detailed reaction rate laws were developed in relatively small‐scale, controlled experimental systems, often well‐mixed reactors, where concentrations of all participating chemicals can be measured. Therefore, the use of these rate laws requires concentrations of all involved species. In natural systems, we often do not know the concentrations of all participating chemicals and how they limit each other. We also have limited information on spatial heterogeneities of minerals and substrate materials and which minerals are effectively reacting. We can only infer rates from measured data. As a result, we often have to simplify these rate laws and use relatively crude representations of reaction rates that involve fewer parameters. However, that does not mean Monod rate laws are not useable at the watershed scale. With measured concentrations of electron donors and acceptors at the watershed outlet that reflect “average” watershed‐scale dynamics, we can use Monod rate laws to infer the average redox conditions and biogeochemical reaction sequence at the watershed scale. For this reason, we include the ability to use this law in the model and leave the decision of whether or not they use this capability to users.

### 2.4 Numerical Scheme

The code uses a similar numerical scheme as BioRT‐Flux‐PIHM (Zhi et al., 2022) with some slight modifications. Sequential noniterative approach (SNIA), an operator splitting method that separates transport and reaction processes, was used to solve the equations (Steefel & MacQuarrie, 1996; Walter et al., 1994). Transport was solved using Backward Euler method, while reactions were solved iteratively using Crank‐Nicholson and Newton‐Raphson method with adaptive time steps (time step was halved iteratively till solution convergence). The system of linear equations derived from the discretization of ordinary differential equations (ODE) at each time step was solved using CVODE, a numerical ODE solver in SUite of Nonlinear and Differential/ALgebraic equation Solvers (SUNDIALS) (Hindmarsh et al., 2005).

### 2.5 Model Setup and Input/Output

**Figure 2:**
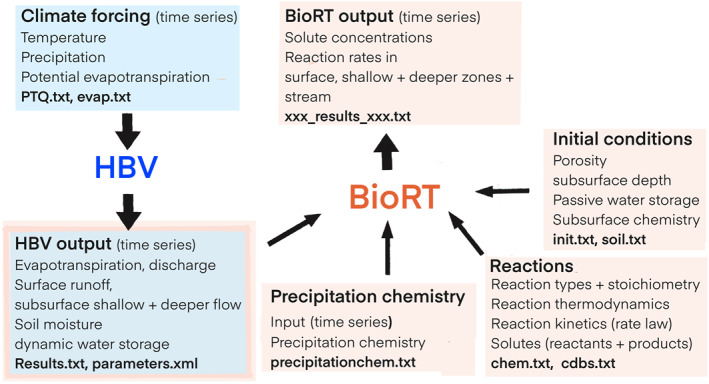
The model structure of BioRT‐HBV. HBV (blue boxes) is run first, followed by BioRT‐HBV (pink boxes). HBV requires time series of climate and weather forcing data (air temperature, precipitation, and potential evapotranspiration); HBV outputs time series of water storage and flows (blue boxes wrapped in pink frame in bottom left). The model output and parameter files of HBV are used as input for BioRT‐HBV, along with other input files that describe the chemical forcing (precipitation chemistry), reaction network, and initial conditions. BioRT‐HBV output includes time series of solute concentrations in different zones and in stream along with reaction rates in different zones.

The model structure and setup of BioRT‐HBV is shown in Figure 2. The input of HBV includes time series of precipitation, temperature, potential evapotranspiration and stream discharge (for calibration) (Seibert, 2005). HBV can be calibrated either manually or using automatic methods like genetic algorithm optimization (Seibert, 2000; Seibert & Vis, 2012; Vis et al., 2015). Monte Carlo simulations have also been used to identify cases that reproduce streamflow data (Sadayappan et al., 2023). Modeled water fluxes and storage from HBV along with its parameters are used as hydrological inputs for BioRT‐HBV.

Depending on research needs, users can prescribe the complexity of the reaction network, including the type and number of reactions, and the number of solutes and sediments to be included in the input files. Reaction stoichiometry, thermodynamics, and kinetics can be specified in the geochemical database and input files. The database follows the format of the CrunchFlow model database (Steefel, 2009). The model input also includes watershed characteristics including passive water storage, porosity, depths of the different zones as well as initial concentrations of primary species that serve as the building blocks of chemical systems, mineral specific surface area and the dependence functions (soil moisture, temperature and water table depth) for reactions in each zone. Subsurface properties like depth, porosity and surface areas of reacting materials are considered as constant because the time scales considered in BioRT‐HBV span from days to years.

The model output includes concentrations of different solutes and sediments in snow, surface zone, shallow zone, deep zone, and streams, as well as rates of kinetically‐controlled reactions in these zones. BioRT‐HBV can be run at flexible time steps, as long as the time steps are regularly spaced and consistent across input files and in both models. It also needs to be specified in the input file of BioRT‐HBV. For example, if the time series of temperature and precipitation of HBV inputs are at the daily scale, the precipitation chemistry timeseries in the BioRT‐HBV input file should also be at the daily time scale. The output of BioRT‐HBV will also be correspondingly at the daily scale. If instead the precipitation and all other inputs are at minute or hourly scales, the output will be at the corresponding time scale.

## 3 Example Model Applications

BioRT‐HBV has been used to understand reactive transport processes in multiple watersheds, including the catchments W‐9 in the Sleepers River Research Watershed in Vermont (Stewart et al., 2024), and Coal Creek in Colorado (Kerins et al., 2024). Here we showcase the application of BioRT‐HBV in W‐9 in Vermont, US. We first describe W‐9 catchment and its hydrology to provide context, followed by the simulation of carbon and nitrogen processes. Carbon processes modeled include soil respiration (lumped heterotrophic and autotrophic respiration) that produces Dissolved Organic Carbon (DOC) and Dissolved Inorganic Carbon (DIC), adsorption of DOC to soils, and carbonate weathering. Nitrogen (N) processes include N leaching, plant N uptake, and denitrification. The carbon processes were calibrated using stream chemistry, whereas the nitrogen processes are presented as an uncalibrated example. These reaction examples are not meant to be comprehensive but serve the purpose of illustrating what can be done using BioRT‐HBV.

### 3.1 Study Site

**Figure 3:**
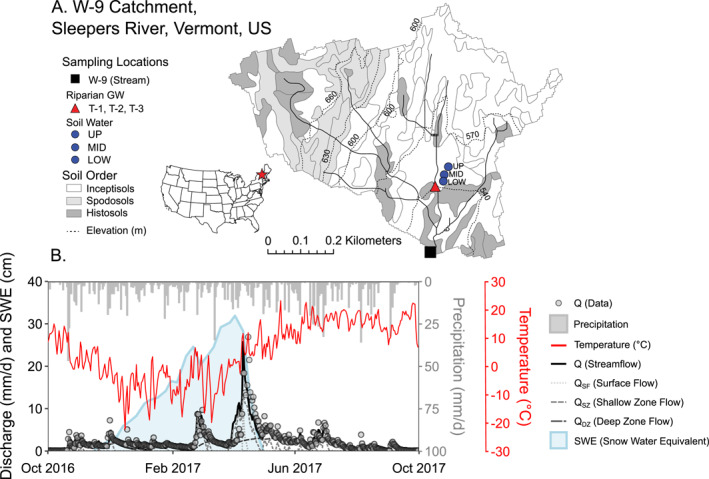
(a) Location of W‐9 catchment in Sleepers River, Vermont, US and its water sampling locations; (b) Time series of observed precipitation (mm/day), temperature (°C), discharge (mm/day), snow water equivalent (mm/day, SWE), HBV model flow outputs (mm/day, lines) and observed stream discharge data (mm/day, dots) at W‐9 for water year 2017.

W‐9 is a small, forested headwater catchment (0.405 km^2^) in the Sleepers River Research Watershed in northeastern Vermont, USA (Figure 3a). It has a humid continental climate with mean annual precipitation of 1,320 mm and mean annual temperature of 4–6°C (Armfield et al., 2019; Sebestyen et al., 2009). Approximately 20%–30% of annual precipitation falls as snow; 40% of precipitation is partitioned to evapotranspiration, while the remaining 60% is partitioned to stream runoff (Shanley, 2000). Soils on hillslopes are well‐drained inceptisols and spodosols while riparian soils are poorly drained histosols. The catchment is underlain by quartz‐mica schist and carbonate‐containing calcareous granulite bedrock (Shanley, 2000; Shanley et al., 2004). Weathering of carbonate minerals (mostly calcite) produces base cations like calcium (Ca) and carbonate species, resulting in well‐buffered subsurface and stream water (Adler et al., 2021; Shanley, 2000). W‐9 catchment was represented in BioRT‐HBV as a single grid with two subsurface zones (SZ and DZ) (Figure S1 in Supporting Information S1).

We used hydrometeorology and stream chemistry data from W‐9 for model set‐up and calibration. Hydrometeorology data, including daily precipitation, temperature, and discharge, and ∼weekly stream chemistry (DOC, DIC, Ca) data were measured at W‐9 by the U.S. Geological Survey from 1991 to 2018 (Matt et al., 2021; Shanley et al., 2021). Daily potential evapotranspiration was not measured at W‐9, so values from the nearby Hubbard Brook Experimental Forest in New Hampshire were used as a proxy (Green et al., 2021).

W‐9 is seasonally snow‐dominated, with discharge often peaking in March or April following spring snowmelt (Figure 3b). Model output from HBV suggests that annual discharge is dominated by deep flow *Q*_DZ_ (∼64%), followed by shallow flow *Q*_SZ_ (∼35%) and minimal contributions from *Q*_SF_ (∼1%). Most *Q*_SF_ occurs during snowmelt. At the daily scale, shallow flow *Q*_SZ_ often dominates under wet conditions (e.g., spring snowmelt) and *Q*_DZ_ dominates under dry conditions (e.g., summer). This partitioning of discharge is consistent with previous studies that highlight a strong groundwater signature in baseflow stream chemistry and new water contributions through surficial soils and shallow flow paths following events like snowmelt and storms (Kendall et al., 1999; Sebestyen et al., 2008; Shanley et al., 2002, 2015).

### 3.2 Carbon Processes

#### 3.2.1 Reaction Network

To simulate the dissolved carbon dynamics in stream and understand the subsurface biogeochemical processes controlling them, we include both carbonate weathering (carbonate lithology at W‐9) and soil respiration, including heterotrophic respiration (decomposition of organic carbon) by microbes and autotrophic respiration by plant roots, among other reactions in the model (Table 1). Three reactions included to capture DOC dynamics are shallow zone soil respiration (Resp_SZ_), deep zone respiration (Resp_DZ_), and equilibrium‐controlled sorption in the shallow zone (Sorption_SZ_). Resp_SZ_ represents the net DOC and DIC production from microbial processing of soil organic carbon (OC_SZ_) and root respiration and exudates (Roots_DOC&DIC_). While DOC can be generated via SOC decomposition and root exudation in the soil, it can also get decomposed by microbes. DOC accumulates only when its generation rate exceeds decomposition rate. However, we do not have data to differentiate these individual reaction pathways. We therefore use this broad, umbrella reaction (Resp_SZ_) to represent the net reaction in the SZ. While OC_SZ_ content can vary over time, here we do not simulate the dynamics of OC_SZ_ as our focus is on simulating net DOC production rates. We assume that the forested catchments like Sleepers River always have abundant soil organic carbon to not limit DOC production. Resp_DZ_ represents the decomposition of translocated DOC from the shallow zone and petrogenic carbon into DIC in the DZ (Dean, 2019; Soulet et al., 2018). Deep root exudates (Tune et al., 2020) can also act as sources of DIC. We use the temperature dependence function (*f*(*T*) in Equation 5) to account for the generally higher respiration rates in the summer, and the soil moisture function (*f*(*S*_*w*_) in Equation 6) to account for rate dependence on water content. These reactions are considered as occurring only in the shallow and deep zones, as their occurrence at the ground surface tends to be minimal due to fast runoff and short contact time with surface materials. DOC produced in the SZ can sorb on soil mineral surfaces (sorption sites ≡X) and become temporarily immobilized as ≡XDOC. Sorption is not included in the DZ as it has negligible sorption sites.

**Table 1:** Reaction Network and Parameters for BioRT‐HBV Model Calibrated for Respiration and Weathering in W‐9 Catchment of Sleepers River

Reactions	Rate law	log_10_ Keq	log_10_ *k* (mol/m^2^/s)	SSA (m^2^/g)	f(T) *Q*_10_	$f\left(S_{w}\right)$ *n*, *S*_*w,c*_	$f\left(Z_{w}\right)$ *α* × *β*
***Shallow Zone Reactions***							
***(1) Respiration (Resp***_***SZ***_ ***):*** $\text{OC}\left(s\right)+{\text{Roots}}_{\text{DOC}\;\&\;\text{DIC}}\to 0.6\text{DOC}+0.55\text{DIC}$	$r_{1}=kA\prod_{ii=1}^{mm}\left(\frac{C_{ii}}{C_{ii}+K_{M,ii}\;}\right)$ *K*_*M,OC (s)*_ = 6 × 10^−6^ mol/L^a^	NA	−10.2	0.10	2.30	0.8, 0.7	0
***(2) Carbonate***_***SZ***_: ${\;\text{Carbonate}}_{\text{SZ}}\left(s\right)\to 1.1{Ca}^{2+}+0.5DIC$	$r_{2}=kA\left(1-\frac{IAP}{K_{eq}}\right)$	−7.40	−9.19	1.00	1.00	1.0, 1.0	0
***(3) Sorption (Sorption***_***SZ***_ ***):*** ≡X+DOC↔≡XDOC	Equilibriumreaction	−1.00	NA	1.0	NA	NA	NA
***(4) CO***_***2***_ ***Gas – Aqueous Exchange:*** ${CO}_{2}\left(*g\right)\;↔\;{CO}_{2}\left(aq\right)$	$r_{4}=kA\left(1-\frac{IAP}{K_{eq}}\right)$	−3.20	−13.10	0.01	3.0	2, 0.7	0
***Deep Zone Reactions***							
***(5) Resp***_***DZ***_ ***:*** ${OC}_{DZ}\left(s\right)+DOC\;\to 0.7DIC$	$r_{5}=kA\left(\frac{C_{ii}}{C_{ii}+K_{M,ii}\;}\right)$ *K*_*M,*DOC_ = 5 × 10^−3 ^mol/L^b^	NA	−9.2	0.07	1.00	1.2, 0.6	0
***(6) Carbonate***_***DZ:***_ ${\;\text{Carbonate}}_{\text{DZ}}\left(s\right)\to 0.9{Ca}^{2+}+0.7\text{DIC}$	$r_{6}=kA\left(1-\frac{IAP}{K_{eq}}\right)$	−7.40	−9.19	0.0008	3.00	0.9, 1.0	0
***(7) CO***_***2***_ ***Gas – Aqueous Exchange:*** ${CO}_{2}\left(*g\right)\;↔\;{CO}_{2}\left(aq\right)$	$r_{7}=kA\left(1-\frac{IAP}{K_{eq}}\right)$	−3.20	−13.10	0.007	1.5	0, 0.7	0
***Equilibrium Reactions in both Shallow and Deep Zones***							
***Carbon Speciation Reactions***							
***(8)*** ${CO}_{2}\left(aq\right)+H_{2}O\;↔$ ${HCO}_{3}^{-}+H^{+}$	Equilibriumreaction	−6.35	NA	NA	NA	NA	NA
***(9)*** ${HCO}_{3}^{-}\;↔\;{CO}_{3}^{2-}+H^{+}$	Equilibriumreaction	−10.33	NA	NA	NA	NA	NA

Carbonate weathering reactions, Carbonate_SZ_ and Carbonate_DZ_, represent the dissolution of carbonate‐containing minerals to produce calcium ions (Ca^2+^) and DIC (via CO_3_^2−^) following the TST rate law. Carbonate_DZ_ follows the same reaction as Carbonate_SZ_, though reaction rates and stoichiometry differ due to differences in the origin and composition of carbonate in the two zones. The shallow zone carbonate is usually pedogenic carbonate generated by carbonate precipitation in soil under dry conditions (Macpherson & Sullivan, 2019; Zamanian et al., 2016), whereas the deep zone carbonate is typically partially weathered or unweathered carbonate bedrock.

DIC is the sum of all dissolved inorganic carbon species (CO_2_(aq), HCO_3_^−^ and CO_3_^2−^). The model simulates carbonate speciation reactions between these three species, depending on pH. High concentration of CO_2_(aq) leads to the formation of soil CO_2_. The extent of this CO_2_ gas‐aqueous exchange is determined by CO_2_ solubility prescribed by Henry's Law. In the model, we represent CO_2_(*g) as an immobile pseudo‐gas phase that can dissolve and become CO_2_(aq), which can further speciate to become HCO_3_^−^ and CO_3_^2−^ under different pH conditions. DIC production and CO_2_ gas‐aqueous exchange are coupled processes, so they are typically simulated together.

Model parameters were manually calibrated to stream chemistry observations for water years 2016–17, representing two consecutive years with sufficient data and distinct discharge dynamics (i.e., small and large snowmelt events). We did not include a validation period because the model is not used to predict future behavior, only to understand the processes behind observed stream discharge and chemistry dynamics. However, using the model parameters to predict stream chemistry in a later year (2018) yielded satisfactory performance (NSE values > 0.4). A Monte Carlo analysis revealed that the manually calibrated parameters yielded the best performance across a range of HBV cases. Calibration procedure is detailed in Stewart et al. (2024) and is reproduced in the SI (“Text S1 in Supporting Information S1: Model calibration and Monte‐Carlo analysis for carbon processes at W‐9”). Here, we show results for water year 2017 for simplicity. The model performance metrics are summarized in Table S1 in Supporting Information S1. The full reaction network and calibrated parameters are summarized in Table 1.

SSA – Specific Surface Area of mineral (m^2^/g); (a) – OC(s) was used as Monod term for respiration in the SZ. Monod term value is low as OC(s) is abundant enough to not limit DOC production; (b) – DOC was the Monod term for respiration in the DZ. *The gas‐aqueous exchange represents the exchange reactions between soil CO_2_ (CO_2_(*g) and dissolved CO_2_ (CO_2(aq)_). NA means the parameter is not applicable for the particular reaction network.

#### 3.2.2 Reactive Transport Equations

In this particular example, the five primary species are DOC, HCO_3_^−^, Ca^2+^, H^+^, and ≡X; the secondary species are CO_3_^2−^, CO_2_(aq), OH^−^ and ≡XDOC. Dissolved inorganic carbon (DIC) is the summation of CO_2_(aq), HCO_3_^−^ and CO_3_^2−^. The model solves Equations (1), (2), (3) for the concentrations of all five primary species. The concentrations of secondary species are expressed via equilibrium relationships after time stepping for the primary species. The overall reaction rate *R* (i.e., *R*_SF_, *R*_SZ_, *R*_DZ_ in Equations (1), (2), (3)) for each primary species could be the summation of multiple rates from different reactions. For example, Ca^2+^ is only involved in carbonate weathering, such that the *R*_SZ_ and *R*_DZ_ terms in its Equations 2 and 3 only include rate expressions for reactions (Equation 2) and (Equation 6) and the corresponding reaction stoichiometry coefficients in Table 1, respectively. In other words, *R*_SZ,Ca_ = *α*_2,Ca_ *r*_2_ = 1.1 *r*_2,_ and *R*_DZ,Ca_ = *α*_6,Ca_ *r*_2_ = 0.9 *r*_6._ The *α* values in front of *r* refer to the reaction stoichiometry coefficients corresponding to the species specified in the “Reactions” column in Table 1. In contrast, HCO_3_^−^ and other inorganic carbon forms are involved in multiple reactions, such that *R*_SZ, HCO3−_ = *α*_1_ *r*_1_ + *α*_2_ *r*_2_ + *α*_4_ *r*_4_, as these three kinetic reactions together contribute to the production of DIC. Similarly, *R*_DZ, HCO3−_ = *α*_5_ *r*_5_ + *α*_6_ *r*_6_ + *α*_7_ *r*_7._ Note that the model ensures unit consistency while solving these equations.

#### 3.2.3 Interrogating Model With Data to Understand Influential Reactions

**Figure 4:**
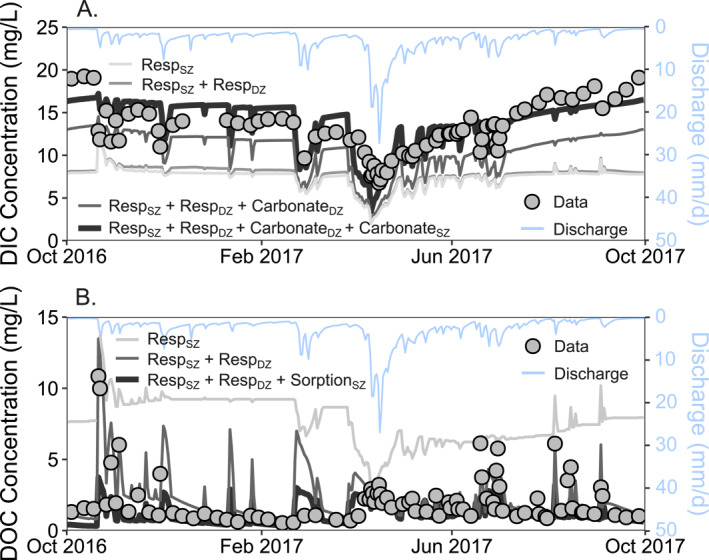
BioRT‐HBV model output of stream concentrations (mg/L) for (a) Dissolved Inorganic Carbon (DIC) and (b) Dissolved Organic Carbon (DOC). The different lines are different model outputs when including different reactions (Resp_SZ_, Resp_DZ_, Sorption_SZ_, Carbonate_SZ_, and Carbonate_DZ_); the lines are compared to data (dots) to illustrate how much they capture the dynamics in data. Stream DIC concentrations are only reproduced by the model when all reactions are included, indicating the importance of both carbonate weathering and respiration sources. DOC is primarily produced via soil respiration (Resp_SZ_) and consumed via deep respiration (Resp_DZ_). The model without deep respiration overestimates DOC and produces opposite trend of DOC from data (low DOC at high discharge), underscoring the importance of deep respiration. The sorption reaction (Sorption_SZ_) acts as a buffer and reduces the overall stream concentrations of DOC, particularly peak concentrations.

Stream chemistry reflects the influence of multiple reactions such that it is often challenging to differentiate the role of individual reactions. Here we illustrate that the model can be used to distinguish the role of different reactions in determining stream solute dynamics (Figure 4a). For example, when Resp_SZ_ is the only reaction included in the simulation, modeled DOC concentrations are high throughout the year, rather than only at high flow conditions as observed in data. Adding deep respiration Resp_DZ_ reduces the low flow concentrations of DOC as the deep zone reaction consumes DOC translocating from the shallow zone, thereby reducing DOC concentrations in the DZ and bringing the modeled DOC closer to data. This indicates the importance of deep zone DOC consumption. Stream DOC is also influenced by sorption of DOC onto soils in the SZ (Sorption_SZ_). Sorption is more likely to occur in the SZ due to higher clay content. Sorption stores some of the produced DOC on soil surface such that not all DOC is flushed to the stream at high discharge. In other words, some DOC is retained and stored on soils in the SZ, which is consistent with field observations (Neff & Asner, 2001) and observations in other places (Wen et al., 2020). When only including respiration, modeled DIC concentrations in the stream are lower than measured DIC, regardless of Resp_SZ_ model parameters, indicating the presence of an additional DIC source (Figure 4b). When carbonate weathering is included as well, stream DIC concentrations from the model agree better with data, suggesting that both biogenic and geogenic sources contribute to stream DIC. The calibrated model occasionally produces spikes and drops that are not observed in data. This difference may be due to the mismatch between sampling frequency of chemistry data and the daily time step of the model, such that the model exhibits more dynamic behavior than what we observe in weekly stream chemistry data.

#### 3.2.4 Understanding Patterns of Dissolved Carbon Concentrations in the Subsurface and Stream

**Figure 5:**
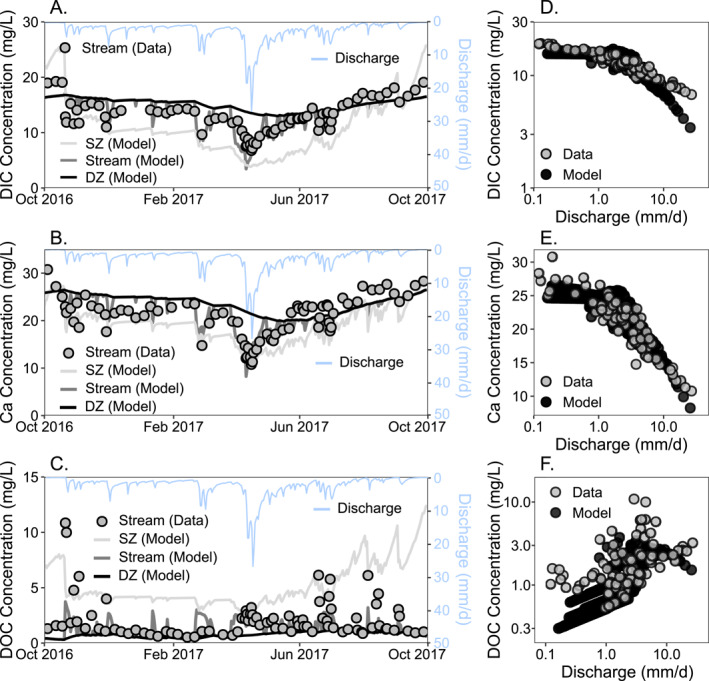
Time series of data and modeled concentrations (mg/L) in stream, shallow zone (SZ), and deep zone (DZ) for (a) Dissolved Inorganic Carbon (DIC), (b) Calcium (Ca), (c) Dissolved Organic Carbon (DOC). Concentration‐discharge plots for stream chemistry data and model output for (d) DIC, (e) Ca, (f) DOC.

The modeled stream concentrations (Figure 5) show that the calibrated model can reproduce the temporal dynamics and Concentration‐Discharge (CQ) patterns of DIC, DOC, and Ca well considering the relatively low complexity of the model. The calibrated model can be used to understand carbon processes on land and in streams. Simulation outputs show that the concentrations of DIC and Calcium (Ca) are higher in the DZ than the SZ. Correspondingly, stream DIC and Ca concentrations at low flow conditions dominated by groundwater are generally high. During high discharge, stream DIC and Ca are diluted by the large input of shallow soil water with low Ca and DIC, as demonstrated in the negative, or dilution, relationships between concentration and discharge for both solutes. Concentrations of DOC are lower in the DZ than in the SZ, such that stream concentrations are low when DZ water dominates at low discharge and increase with discharge as *Q*_SZ_ from the SZ increasingly contributes to streamflow. This leads to a positive, or flushing, relationship between DOC concentrations and discharge. Stream DIC and Ca concentrations are relatively stable compared to DOC but exhibit a more pronounced seasonality with lowest concentrations in early spring (∼April) and highest concentrations in late summer (∼September). Note that these dynamics arise from the integrated effects of biogeochemical reactions in the subsurface and the flow partitioning. While the results shown here represent one BioRT‐HBV case, a Monte Carlo analysis revealed that the discharge partitioning from HBV had to be in a narrow range (1%–5% *Q*_SF_, 25%–34% *Q*_SZ_, and 64%–69% *Q*_DZ_) to reproduce stream chemistry dynamics (Refer to Text S1 in Supporting Information S1, adapted from Stewart et al. (2024)).

#### 3.2.5 Understanding the Spatial and Temporal Variation of Carbon Production and Export

**Figure 6:**
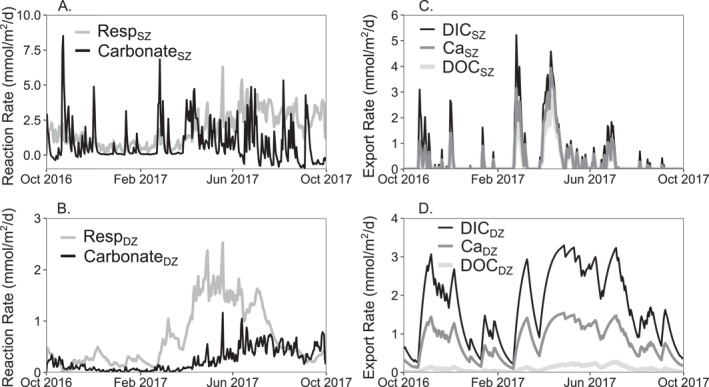
Time series of modeled reaction rates (mmol/m^2^/day) in (a) shallow zone (SZ) and (b) deep zone (DZ); Time series of export rates (mmol/m^2^/day) for Ca, DIC, and DOC from (c) SZ and (d) DZ. Reactions include Resp_SZ_, Carbonate_SZ_, Resp_DZ_, and Carbonate_DZ_.

In addition to understanding the processes that regulate stream chemistry, the calibrated model can also be used to understand the temporal variations in reaction rates and solute export patterns (Figure 6). The model simulation shows that the carbonate weathering rates in the SZ (Carbonate_SZ_) vary considerably (Figure 6). Carbonate_SZ_ has a baseline rate of 0, indicating that the carbonate mineral in the SZ is generally at equilibrium with water. The reaction rate can increase to positive values indicating dissolution, or decrease to negative values indicating calcite precipitation (mostly in summer months at low discharge). The soil respiration rate (Resp_SZ_) shows a strong seasonality, with highest rates in warm, summer months and lowest rates in cool, winter months. In the deep zone, rates are lower overall; Carbonate_DZ_ exhibits less flashy dynamics than Carbonate_SZ_ and a similar seasonal pattern as Resp_SZ_ with higher rates in summer months and lower rates in winter months. Deep respiration (Resp_DZ_) rates, however, show more drastic seasonal behavior with highest rates in spring and lowest rates in autumn and late winter. This is because high flow in spring leads to high recharge and more transport of DOC to the DZ, facilitating deep respiration. Export rates from the SZ are flashy and peak during high discharge, while export rates from the DZ are more stable due to steady contributions of groundwater flow (*Q*_DZ_) to the stream. Ca and DIC are exported from both subsurface zones, though export from the DZ is more persistent. DOC is primarily exported from the SZ; DOC export from the DZ is negligible.

The reaction rates discussed here are outputs from the calibrated model, as reaction rates, especially those that occur in the subsurface, are not directly measurable. Reaction rates are generally inferred from time series of concentration measurements (Brantley et al., 2023; White & Brantley, 2003). It is important to note that the rates quantified via this bucket watershed‐scale model represent average rates at the watershed scale that are rarely quantified compared to rates derived from controlled lab experiments and those inferred from field studies (White & Brantley, 2003). As only major processes controlling solute behavior are simulated, modeled solute export dynamics might only be capturing the general patterns of solute behavior and not their finer details.

The model results echo previous studies that highlight the importance of vertical distribution of solute concentrations in the subsurface and the shifting dominance of deeper, slower source waters to shallower, faster source waters under low to high flow conditions, in determining stream chemistry variations and solute exports, also known as the Shallow and Deep hypothesis (Stewart, Shanley, et al., 2022; Wen et al., 2022; Xiao et al., 2021; Zhi et al., 2019). The model offers additional insights on where, when and at what rates biogeochemical reactions occur, compared to insights inferred direct from measurement data without reactive transport modeling (Keller, 2019; Tune et al., 2020).

### 3.3 Nitrogen Processes

#### 3.3.1 Reaction Network

BioRT‐HBV can also be used to explore the role of different reactions in determining concentrations, rates, and stream chemistry dynamics in numerical experiments. Here we demonstrate such capability using processes related to nitrate for Sleepers River. Nitrate is an essential solute in nitrogen biogeochemical cycle which can contribute to greenhouse gas emissions and cause widespread eutrophication and hypoxia (Ma et al., 2023; Sadayappan et al., 2022; Van Meter et al., 2018). For simplicity, we include three major biogeochemical reactions that produce and consume nitrate (NO_3_^−^): soil nitrogen (N) leaching, plant uptake of NO_3_^−^, and denitrification (Table 2). Soil N leaching is a lumped reaction that represents the net nitrate production from soil organic matter decomposition, nitrification, and rock weathering processes that generate NO_3_^−^. Plant uptake is represented by a simple approach that accounts for NO_3_^−^ removal from soil by plant assimilation. The denitrification reaction is represented with NO_3_^−^ being fully reduced to N_2_O although NO_3_^−^ can also be reduced to N_2_ and a suite of other N‐containing solutes. These reactions follow the simplified rate law $r=kAf\left(T\right)f\left(S_{w}\right)f\left(Z_{w}\right)$ (Equation 4), without explicitly considering dependence on other solutes. Parameters for the nitrogen reaction network (Table 2) were manually adjusted to produce the commonly observed flushing pattern for nitrate, but parameters were not calibrated to observed stream concentrations.

**Table 2:** Reaction Network and Parameters for Nitrate Processes in BioRT‐HBV Model

Reactions	Rate law	log_10_ *k* (mol/m^2^/s)	SSA(m^2^/g)	f(T) *Q*_10_	$f\left(S_{w}\right)$ *n*, *S*_*w,c*_	$f\left(Z_{w}\right)$ *α* × *β*
***Shallow Zone Reactions***						
***Nitrogen Leaching (NLeaching***_***SZ***_ ***):*** $soilN\;\to NO_{3}^{-}$	$r=kA\prod_{ii=1}^{mm}\left(\frac{C_{ii}}{C_{ii}+K_{M,ii}\;}\right)$ *K*_*M,*soilN_ = 6 × 10^−4^ mol/L^a^	−15.0	5.5	2.0	1, 1.7	0
***Plant Uptake (PlantUptake***_***SZ***_ ***):*** $NO_{3}^{-}\;\to PlantN$	r=kA	−13.4	3.0	1.5	1, 1.25	0
***Denitrification (Denitrification***_***SZ***_ ***):*** $NO_{3}^{-}\;\to \;N_{2}O$	$r=kA\prod_{ii=1}^{mm}\left(\frac{C_{ii}}{C_{ii}+K_{M,ii}\;}\right)$ *K*_*M*, NO3−_ = 1 × 10^−6^ mol/L^b^	−12.8	1 × 10^−6^	1.0	1, 0	0
***Deep Zone Reactions***						
***NLeaching***_***DZ***_ ***:*** $soilN\;\to NO_{3}^{-}$	$r=kA\prod_{ii=1}^{mm}\left(\frac{C_{ii}}{C_{ii}+K_{M,ii}\;}\right)$ *K*_*M,*soilN_ = 6 × 10^−4^ mol/L^a^	−15.0	1 × 10^−4^	1.0	1, 0	0
***PlantUptake***_***DZ***_ ***:*** $NO_{3}^{-}\;\to PlantN$	r=kA	−13.4	1 × 10^−4^	2.5	1, 0	0
***Denitrification***_***DZ***_ ***:*** $NO_{3}^{-}\;\to \;N_{2}O$	$r=kA\prod_{ii=1}^{mm}\left(\frac{C_{ii}}{C_{ii}+K_{M,ii}\;}\right)$ *K*_M, NO3−_ = 1 × 10^−6^ mol/L^b^	−12.8	3 × 10^−4^	1.5	1, 0	0

*Note.* SSA – Specific Surface Area of mineral (m^2^/g).

^a^ soilN was the Monod term for leaching.

^b^ NO_3_^−^ was the Monod term for denitrification.

**Figure 7:**
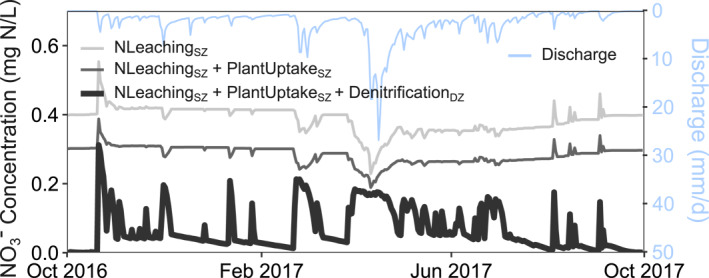
BioRT‐HBV model output of Nitrate (NO_3_^−^) concentrations (mg/L) with different reaction combinations. NO_3_^−^ is produced by NLeaching in the shallow zone (SZ), but typical stream NO_3_^−^ concentrations and dynamics are only reproduced when both PlantUptake_SZ_ and Denitrification_DZ_ reactions are included to consume NO_3_^−^ in the SZ and deep zone (DZ) respectively. Note that the inclusion of Denitrification_DZ_ (darkest gray) led to a pattern opposite from those excluding this reaction (lighter grays).

The modeled nitrate concentrations in Figure 7 illustrates the role of different types of reactions in determining stream nitrate dynamics. When only N Leaching reaction in the SZ (NLeaching_SZ_) is simulated, concentrations are generally higher than other cases and show a dilution pattern (higher concentrations at low discharge conditions). Adding the plant uptake reaction reduces stream NO_3_^−^ concentrations, but the temporal dynamics of stream NO_3_^−^ remains similar to the case with only NLeaching_SZ_. The dynamics change substantially when denitrification reaction is included in the DZ (Denitrification_DZ_), resulting in lower concentrations during low flow but higher concentrations during high flow.

#### 3.3.2 Nitrate Concentration Dynamics, Reaction Rates and Export Dynamics

**Figure 8:**
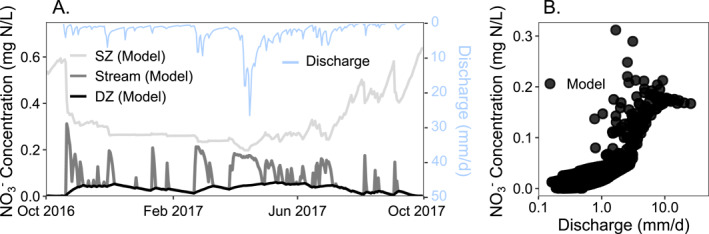
(a) Time series of modeled nitrate (NO_3_^−^) concentrations (mg/L) in stream, shallow zone (SZ), and deep zone (DZ). (b) Corresponding concentration‐discharge plot for modeled stream NO_3_^−^.

**Figure 9:**
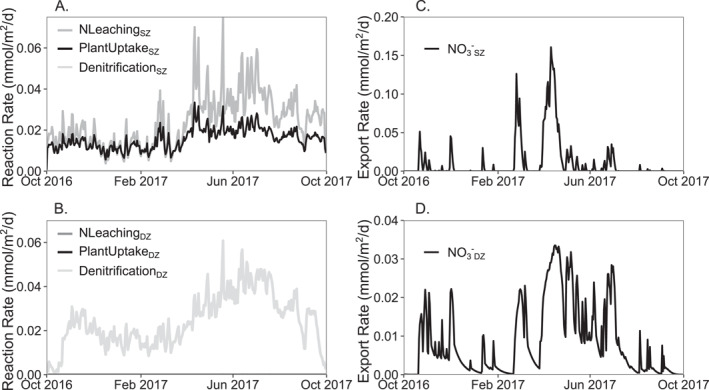
Time series of overall reaction rates (mmol/m^2^/day) for A. shallow zone (SZ) and B. deep zone (DZ) for NLeaching_SZ_ (nitrate leaching), PlantUptake_SZ_ (plant assimilation of nitrate), and Denitrification_DZ_. Rates of Denitrification_SZ_, NLeaching_DZ_, and PlantUptake_DZ_, are effectively zero. Time series of overall export rates (mmol/m^2^/day) for Nitrate (NO_3_^−^) from the C. SZ and D. DZ.

The model output in the case with all three reactions (NLeaching_SZ_, PlantUptake_SZ_, and Denitrification_DZ_ for NO_3_^−^) shows high nitrate concentrations in the SZ where the N leaching rate exceeds the plant N uptake rate, and low nitrate concentrations in the DZ due to denitrification (Figures 8a and 9a). As a result, high stream nitrate concentrations occur when *Q*_SZ_ contributes to the stream. This leads to a flushing concentration‐discharge relationship (Figure 8b) that echoes the typical flushing pattern seen in observed data (Porter et al., 2022; Stewart, Shanley, et al., 2022). This highlights the need for considering denitrification processes in the subsurface to capture observed stream NO_3_^−^ dynamics.

Nitrate leaching and plant uptake mostly occur in the SZ, whereas denitrification mostly occurs in the DZ (Figure 9). NLeaching_SZ_ rates are generally lower in autumn and winter and higher in spring and summer, peaking in April and May (Figure 9a). PlantUptake_SZ_ rates follow a similar pattern as NLeaching_SZ_ but exhibit a smaller increase in spring and summer months. Denitrification_DZ_ rates show similar seasonal dynamics as NLeaching_SZ_ and PlantUptake_SZ_ with lower rates in autumn and winter and higher rates in spring and summer. Hydrology and the temporal changes in relative contribution of flow paths determine nitrate export dynamics (Figures 9c and 9d) with high and low export rates occurring during high, *Q*_SZ_ dominated, and low, *Q*_DZ_ dominated, flow times respectively. Nitrate export occurs mainly from the SZ, while the DZ acts as a smaller but steadier source of nitrate to the stream. These insights align with other studies that explore the reaction and transport pathways of nitrate (Husic et al., 2019).

## 4 Discussion

Here we introduce BioRT‐HBV, a watershed‐scale RTM that can simulate surface and subsurface flow paths and biogeochemical processes. BioRT‐HBV builds upon the widely used HBV hydrology model, inheriting its parsimonious framework. Additionally, it utilizes the traditional framework of RTMs to flexibly and adaptively (to needs of the users) represent a variety of user‐defined biogeochemical reactions. This differentiates it from water quality models that typically model only select predefined solutes (Abdelnour et al., 2013; Hou et al., 2021; Rajib et al., 2020; Veinbergs et al., 2017).

BioRT‐HBV can be a valuable research and educational tool, as demonstrated by the success of HBV‐light (Seibert & Bergström, 2022). An important, often overlooked aspect of models is their accessibility, especially in terms of the user's experience and knowledge. Simple models that are straightforward and easy to understand are more user‐friendly, especially for beginners without in‐depth computational training. Easily accessible models with user‐friendly GUIs can promote their widespread use across different fields, and help foster a more varied and inclusive user community, supporting diversity, equity, and inclusion (Perdrial et al., 2023; Singha et al., 2024).

In the realm of modeling, there has been long‐term debate about the advantages and disadvantages of simple versus complex models (Hrachowitz & Clark, 2017; Wen et al., 2021). Complex models, such as spatially distributed, nonlinear, multi‐component RTMs, can represent and explore the effects of spatial heterogeneities in watershed properties (e.g., soil depth and types, lithology, vegetation, biomass, and mineralogy) on catchment‐scale dynamics including streamflow generation, stream concentration dynamics and solute export patterns (Fatichi et al., 2016; Li et al., 2021). These models are however computationally demanding and present difficulties for ensemble‐based analysis. They also have large number of parameters that lead to issues related to equifinality and uncertainty (Beven, 2006; Beven & Freer, 2001; Kirchner et al., 1996). In recent years, there have been substantial advances in gridded data availability and computing resources, resulting in large‐scale implementation of spatially distributed models (McCabe et al., 2017). Workflow and calibration tools have been developed to help users set up and calibrate spatially distributed models (Coon & Shuai, 2022; Rouholahnejad et al., 2012). Recent work has additionally shown that using input data of high spatial resolution on hydrometeorology, topography, soil, etc. can reduce the need for calibration in spatially distributed hydrological models (Bhanja et al., 2023). The possibility of doing so for RTMs however is unknown and could be challenging. This is because of the myriad of subsurface biogeochemical characteristics needed for RTMs including soil and bedrock biogeochemistry which often require labor intensive plot‐scale measurements. In addition, reactivity of materials such as SOC and rock minerals do not merely depend on their material abundance and cannot be directly inferred, even from intensive data.

Parsimonious models can overcome some of these outstanding challenges. It is in this context that we developed the BioRT‐HBV model. This model conceptualizes the catchment as composed of three vertical zones, namely surface zone, shallow soil zone and deep bedrock zone. Considering each zone as completely mixed, the model simulates the advective transport of solutes and their biogeochemical reactions. By adopting a sparse structure, the model reduces the number of parameters needed but ignores the lateral details in landscape structure. It represents the “average” dynamics of water flow and reactions on land and in rivers at the watershed scale that are eventually reflected in the commonly measured water chemistry data measured at the stream outlet. Like other lumped models, this model leverages the functional relationships that emerge at catchment scale from the small‐scale heterogeneities (Hrachowitz & Clark, 2017).

The primary model assumption is that the stream chemistry dynamics in a catchment can be captured by simulating three major flow pathways – surface, shallow soil and deeper groundwater flow pathways – and biogeochemical processes in the surface, shallow and deep zones. Through this assumption, we underscore the importance of vertical structure in determining flow paths and the depth distribution of biogeochemical properties and reactions in regulating stream chemistry. The importance of chemical contrasts between shallow and deep subsurface has been supported by data from individual watersheds to global scale (Kincaid et al., 2024; Stewart, Shanley, et al., 2022; Stewart, Zhi, et al., 2022; Zhi & Li, 2020) as well as modeling studies (Botter et al., 2020; Kerins et al., 2024; Seibert et al., 2009; Stewart et al., 2024; Zhi et al., 2019). The presence of fewer parameters in BioRT‐HBV helps reducing parameter uncertainty and equifinality. When constrained by multiple data types like stream water, groundwater and soil water chemistry, uncertainty can be further minimized. Despite its advantages, simple models such as BioRT‐HBV are meant to complement but not replace spatially explicit models. As only vertical heterogeneity is represented, the model cannot track dynamics of reactions that vary along lateral landscape directions such as groundwater pollutant plumes, and redox zonation, or identify “hot spots” of biogeochemical reactions (Wen et al., 2020). Spatially distributed models are more suitable for exploring such dynamics (Fatichi et al., 2016).

The lumped approach in BioRT‐HBV can accommodate basins with low data availability, and is more accessible to users from diverse backgrounds, such that process‐based models are not limited to a small group of users with extensive modeling and computational experience. Ultimately, the choice of model complexity level depends on research questions that the model is set to answer, available data and resources. At the end, we all need to balance cost and gain when deciding to use a simple or complex model, striving to be “simple but not simplistic” (Beven & Lane, 2019; Höge et al., 2018; Li et al., 2021).

## 5 Conclusion

Watershed‐scale RTMs are useful tools for understanding and predicting the complex interactions of ecohydrological and biogeochemical processes that influence water chemistry and fluxes on land and in rivers. While traditional RTMs have mainly concentrated on subsurface processes, recent advancements have expanded RTMs to encompass interactions between surface and subsurface processes at the watershed scale. These advanced RTMs represent landscape heterogeneity in detail, but are computationally expensive, and requires users to have extensive computational training. There is a growing need for simple, user‐friendly models that can serve the broader ecohydrological and biogeochemical research community, including those without deep computational experience.

To address this, we introduce BioRT‐HBV 1.0, a simple watershed‐scale model that integrates ecohydrological and biogeochemical processes. BioRT‐HBV builds upon the extensively‐used HBV model and uses its conceptual framework and hydrology outputs. BioRT‐HBV simulates various processes such as solute transport and biogeochemical reactions governed by thermodynamics and kinetics, including chemical weathering, soil respiration, and nutrient transformations. This model simplifies catchment‐scale dynamics by representing three major water flow pathways and the biogeochemical reactions in three zones. This paper outlines the model structure, its governing equations and demonstrates example applications simulating carbon and nitrogen processes in a headwater catchment. BioRT‐HBV model has a simple spatially implicit structure and minimal data requirements, yet can simulate a variety of biogeochemical processes that occur in the invisible subsurface. We put forward BioRT‐HBV as an easily accessible tool for students and researchers from diverse backgrounds, irrespective of their computational expertise.

## Supporting information

Supporting Information S1

## Data Availability

BioRT‐HBV 1.0 model is open source (MIT license) and available for download in GitHub at https://github.com/Li‐Reactive‐Water‐Group/BioRT‐HBV/tree/master as well as Zenodo (Sadayappan et al., 2024b). The input files for simulating carbon and nitrogen processes in the W‐9 catchment of Sleepers Rivers (example showcased in the paper) are also available there. The Graphical User Interface (GUI) is licensed under MIT and is available for download at Sadayappan et al. (2024a).
