# Supplementary material for: BioRT‐HBV 1.0: A Biogeochemical Reactive Transport Model at the Watershed Scale
Source: J Adv Model Earth Syst. 2024 Nov 30;16(12):e2024MS004217. doi: 10.1029/2024MS004217 (PMC11608103; doi:10.1029/2024MS004217)
Supplement: Supplementary file 1 — Supporting Information S1 [file JAME-16-0-s001.pdf]

Journal of Advances in Modeling Earth Systems

Supporting Information for

BioRT-HBV 1.0: a Biogeochemical Reactive Transport Model at the Watershed Scale

Kayalvizhi Sadayappan[1], Bryn Stewart[1], Devon Kerins[1], Andrew Vierbicher[1], Wei Zhi[1], Valerie Diana Smykalov[1], Yuning Shi[2], Marc Vis[3], Jan Seibert[3], Li Li[1,*]

[1] Department of Civil and Environmental Engineering, The Pennsylvania State University, University Park, PA, USA

[2] Department of Plant Science, The Pennsylvania State University, University Park, PA, USA

[3] Department of Geography, University of Zurich, Zurich, Switzerland

*Corresponding author: lili@engr.psu.edu

Contents of this file

Text S1

Figure S1

Table S1

Text S1: Model calibration and Monte Carlo analysis for carbon processes at W-9

We calibrated the BioRT-HBV model on two years of data from W-9 (water years 2016-17), representing two consecutive years with sufficient data and distinct discharge dynamics (i.e., small and large snowmelt events). We show results for water year 2017 here for simplicity. We did not consider a validation period because we were not using the model to predict future behavior, only to understand the processes behind observed stream discharge and chemistry dynamics. The model calibration procedure was first used and detailed in Stewart et al. (2024) and is reproduced here for completeness.

The HBV model was first manually calibrated to determine which parameters were more important in shaping discharge dynamics at W-9. We then ran 1,000,000 Monte Carlo simulations with randomly generated parameter sets and assessed model performance with three commonly used metrics: Nash-Sutcliffe Efficiency (NSE), Kling-Gupta Efficiency (KGE), and Non-Parametric Kling-Gupta Efficiency (NPE) (Gupta et al., 2009; Nash & Sutcliffe, 1970; Pool et al., 2018). These metrics can range from -∞ to 1 and closer the value is to 1, better is the model performance. We focus on NSE here for simplicity. The HBV Monte Carlo analysis produced 110 cases with very good performance (NSE > 0.8), but 16 of these cases has unrealistic discharge partitioning for the study catchment (large Q_{SF} and/or Q_{SZ}). We believed it was not worth including these cases in the BioRT calibration procedure, as the results would not be reasonable. Furthermore, the computational demands for running a Monte Carlo analysis in BioRT for each HBV parameter set were infeasible.

The BioRT model was first manually calibrated by adjusting reaction parameters including reaction rate constants ($log_{10}k$), specific surface area (*SSA*) of solid reactants (i.e., OC and Carbonate), temperature dependence via Q_{10}, and soil moisture dependence via *Sw,c* and *n*.

To consider a broad range of BioRT parameter values in conjunction with different hydrological behavior produced by the HBV-light model, we developed a calibration procedure for sequential Monte Carlo sampling. Following manual calibration, we ran a Monte Carlo analysis by pairing BioRT parameter sets with four hydrology cases from HBV that represented the range of hydrologic behavior from the Monte Carlo cases with

best performance (manually calibrated case, and cases with Q_{DZ} contribution near the 25%, 50%, and 75% quartiles for Q_{DZ} contribution of the 110 Monte Carlo cases). For each hydrology case, we ran 5,000 randomly sampled BioRT parameter sets (produced using a Latin Hypercube Sampling technique), but these combinations (20,000 cases total) did not yield a good performance (NSE > 0 for all three solutes, DOC, DIC, and Ca^{2+}).

We then ran an additional Monte Carlo analysis (1,000 BioRT parameter sets) with narrower parameter ranges based on the manually calibrated BioRT parameters. We also ran each HBV case with the original manually calibrated BioRT parameters for comparison. This analysis resulted in 64 cases NSE > 0 for DOC, 14 cases with NSE > 0 for DIC, and 20 cases with NSE > 0 for Ca^{2+}. Only four of these cases had positive NSE values for all three solutes, three of which used the manually calibrated BioRT parameters. Furthermore, only the cases with the manually calibrated BioRT parameters had NSE values greater than 0.3 for all three solutes.

Considering that the HBV cases with good BioRT performance had ranges of Q_{DZ} contributions from 64 to 69%, we ran the 32 HBV Monte Carlo cases with discharge partitioning that fell in the following ranges: 1-5% Q_{SF}, 25-34% Q_{SZ}, and 64-69% Q_{DZ}. With these additional cases, there were seven total cases with NSE > 0.45 for all solutes, including the manually calibrated HBV case.

For simplicity, we show the results from the manually calibrated case only in this work, but this calibration process revealed that the hydrology model (HBV) was far more prone to equifinality issues than the BioRT model. None of the randomly sampled BioRT parameter sets performed as well as the manually calibrated case, and the manually calibrated case required a narrow range of discharge partitioning (64-69% Q_{DZ}) in the HBV model results to reproduce the observed stream chemistry dynamics. Although limited by computational constraints and model structure, we suggest that the equifinality issue observed with HBV (and commonly found with other hydrology models) is partially counterbalanced by the additional constraints from observed stream and subsurface water chemistry. The reason for this is unclear, but we speculate that it may be due to the high interdependence between solute concentrations and various

reaction rate parameters, leading to a very small parameter space that can reproduce observed solute concentration dynamics. The difference in calibration for hydrology and biogeochemistry models was observed in two studies using this model at different catchments, W-9 at Sleepers River in Vermont, shown in this study as an example (Stewart et al., 2024), and Coal Creek in Colorado (Kerins et al., 2024).

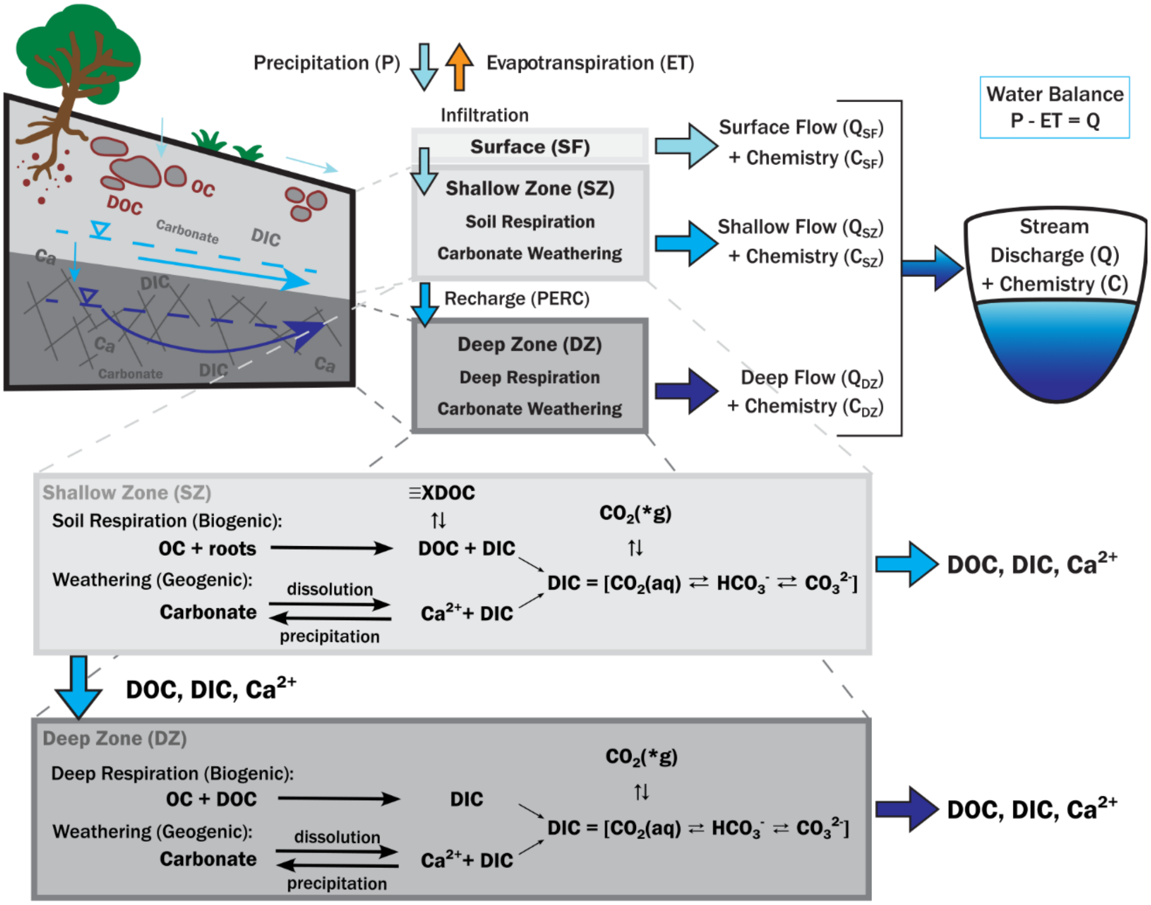

Figure S1 Domain setup of catchment along with reaction network included in BioRT-HBV model. Modified from Stewart et al. (2024).

Table S1. Model performance metrics for the manually calibrated case for simulating carbon processes in W-9 catchment (water years 2016-17)

	**NSE**	**KGE**	**NPE**
**HBV**	0.7	0.84	0.79
**BioRT (DOC)**	0.49	0.57	0.67
**BioRT (DIC)**	0.57	0.79	0.79
**BioRT (Ca)**	0.58	0.77	0.75

References

Gupta, H. V., Kling, H., Yilmaz, K. K., & Martinez, G. F. (2009). Decomposition of the mean squared error and NSE performance criteria: Implications for improving hydrological modelling. *Journal of Hydrology, 377*(1-2), 80-91.

Kerins, D., Sadayappan, K., Zhi, W., Sullivan, P. L., Williams, K. H., Carroll, R. W., et al. (2024). Hydrology outweighs temperature in driving production and export of dissolved carbon in a snowy mountain catchment. *Water Resources Research, 60*(7), e2023WR036077.

Nash, J. E., & Sutcliffe, J. V. (1970). River flow forecasting through conceptual models part I—A discussion of principles. *Journal of Hydrology, 10*(3), 282-290.

Pool, S., Vis, M., & Seibert, J. (2018). Evaluating model performance: towards a non-parametric variant of the Kling-Gupta efficiency. *Hydrological sciences journal, 63*(13-14), 1941-1953.

Stewart, B., Shanley, J. B., Matt, S., Seybold, E. C., Kincaid, D. W., Vierbicher, A., et al. (2024). Illuminating the “Invisible”: Substantial Deep Respiration and Lateral Export of Dissolved Carbon From Beneath Soil. *Water Resources Research, 60*(6), e2023WR035940.
